# Age-Related Changes in MicroRNA in the Rat Pituitary and Potential Role in GH Regulation

**DOI:** 10.3390/ijms19072058

**Published:** 2018-07-15

**Authors:** Haojie Zhang, Qien Qi, Ting Chen, Junyi Luo, Qianyun Xi, Qingyan Jiang, Jiajie Sun, Yongliang Zhang

**Affiliations:** 1 College of Animal Science, South China Agricultural University, Guangzhou 510642, China; zhanghj089@126.com (H.Z.); allinchen@scau.edu.cn (T.C.); junyigzcn@gmail.com (J.L.); xqy0228@163.com (Q.X.); qyjiang@scau.edu.cn (Q.J.); 2 School of Life Science and Engineering, Foshan University, Foshan 528231, China; qiqien1987@163.com; 3 Guangdong Provincial Key Lab of Agro-Animal Genomics and Molecular Breeding, National Engineering Research Center for Breeding Swine Industry, College of Animal Science, South China Agricultural University, Guangzhou 510642, China

**Keywords:** growth hormone, microRNA microarray, pituitary, miR-141-3p

## Abstract

The growth hormone/insulin-like growth factor 1 (GH/IGF-1) axis has recently been recognized as an important factor related to the longevity of many organisms. MicroRNAs (miRNAs or miRs) could also participate in diverse biological processes. However, the role of miRNAs in the decline of pituitary GH during the growth process remains unclear. To better characterize the effects of miRNAs on the pituitary, we used a miRNA microarray to investigate the miRNA profile in the rat pituitary from postnatal development throughout the growth process. Then, in vitro experiments were conducted to analyze the miRNAs’ potential roles related to GH regulation. Taken together, the microarray results indicated that there were 22 miRNAs differentially expressed during pituitary development. The bioinformatics analysis suggested that the most differentially expressed miRNAs may participate in multiple pathways associated with the pituitary function. Furthermore, the in vitro findings demonstrated that miR-141-3p was involved in GH regulation.

## 1. Introduction

The pituitary is a small but complex gland situated beneath the hypothalamus in the sella turcica. It is the “master” endocrine organ that functions as a relay between the hypothalamus and peripheral target organs via five major cell types, including corticotropes, thyrotropes, gonadotropes, somatotropes, and lactotropes [1,2]. Somatotropes specifically produce growth hormone (GH) and comprise the major cell type of the anterior pituitary, constituting approximately 50% of the cell population [1].

GH is one of the major pituitary hormones and the primary regulator of organism growth and metabolism [3,4,5]. Moreover, GH enhances liver insulin-like growth factor 1 (IGF-1) messenger RNA (mRNA) expression [6,7,8] and secretion [9] that mediates a portion of the growth-promoting actions of GH and the GH/IGF-1 axis has recently been recognized as important regarding biological aging and lifespan [10,11,12,13]. The activity of the GH/IGF-1 axis undergoes an age-related reduction [14,15] characterized by a decrease in GH secretion and serum levels of IGF-1 [14,16]. It is possible that changes in nutrition, lifestyle, and alterations in the neurohormonal hypothalamic control of GH secretion might account for the hypoactivity of the GH/IGF-1 axis in aged individuals [17]. However, the molecular mechanisms on the pituitary level remain unclear.

Aging is a natural and inexorable biological process. The changes accompanying aging manifest on both molecular and organismic levels [18]. For example, hormonal systems, particularly the GH/IGF-1 axis, exhibit decreasing circulating hormone concentrations during the process of normal aging [15]. Since aging compromises quality of life, the regulation of aging has drawn great attention. Molecular studies have indicated that microRNAs (miRNAs or miRs) are differentially expressed with advancing age and may play a crucial role in the aging process [19].

MicroRNAs are small noncoding RNAs that play important roles in post-transcriptional gene regulation [20]. In animal cells, miRNAs function by targeting mRNAs for cleavage or translational repression [21]. The expression profiles and function of the pituitary miRNAs are gradually being identified. Moreover, there have been a number of special reports on pituitary miRNAs function. In particular, it was reported that miR-26b targets lymphoid enhancer factor 1 (Lef-1), which upregulates pituitary-specific positive transcription factor 1 (Pit-1) and *GH* expression [22]. miR-375a is a highly expressed miRNA in the pituitary gland and has been reported to regulate the corticotrophin-releasing factor (CRF) signaling pathway and Pro-opiomelanocortin (POMC) expression by targeting mitogen-activated protein kinase-8 (MAP3K8) [23]. Furthermore, several studies have suggested that miRNAs may play a key role in the regulation of gene expression in the heart [24], liver [25], muscle [26], and brain [27] during the aging process; however, the alteration of miRNAs in the pituitary with advancing age remains unclear.

Investigators have identified the differential expression of several miRNAs in various developmental stages in the porcine pituitary, have predicted potential target genes, and defined their biological roles using a gene ontology (GO) analysis [28]. However, it remains necessary and of great significance to explore the effect of pituitary miRNA expression on growth and GH production. Therefore, in the present study, we designed a rat miRNA array and used this method to analyze the miRNA expression in the pituitary of different ages. In addition, the ingenuity pathway analysis (IPA) software and database were used to analyze the 22 miRNAs that exhibited significant changes in expression. We aimed to demonstrate the contribution of altered miRNA expression and multiple signaling pathways involved in the age-related changes that occur in the rat pituitary.

## 2. Results

### 2.1. Rat Growth Curve, Serum IGF-1 Concentration, and Pituitary GH1, Growth Hormone-Releasing Hormone Receptor(GHRHR), and Somatostatin Receptor 2 (SSTR2) mRNA Expression Assay

**Figure 1:**
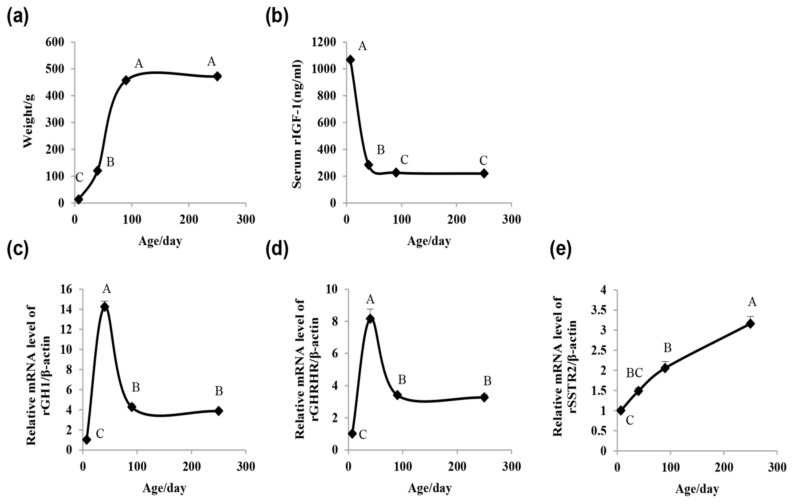
Rat growth curve, serum insulin-like growth factor 1 (IGF-1) concentration, and pituitary growth hormone 1 (*GH1*), growth hormone-releasing hormone receptor (*GHRHR*), and somatostatin receptor 2 (*SSTR2*) messenger RNA (mRNA) expression. (**a**) *Sprague-Dawley* (SD)rats growth curve. (**b**) SD-rat serum IGF-1 concentrations. (**c**–**e**) Alteration of pituitary *GH1*, *GHRHR*, and *SSTR2* mRNA expression. The four points in each figure represent the different ages (Day 7, Day 40, Day 90, and Day 250). The values are expressed as the means ± standard error of the mean (SEM). Means with different letters were significantly different (*n* = 8; *p* < 0.01; ANOVA; Tukey-HSD).

Rat body weight, serum IGF-1 concentration, and pituitary *GH1*, growth hormone-releasing hormone receptor (*GHRHR*), and somatostatin receptor 2 (*SSTR2*) mRNA expression were all markedly age-dependent. Body weight was the lightest on Day 7, increased rapidly until Day 90, and was followed by a gradual reduction in the increasing ratio (Figure 1a). The concentration of serum IGF-1 was the highest on Day 7 and declined rapidly until Day 40, then declined gradually on Day 90 and finally had nearly no alteration on Day 250 (Figure 1b). The level of pituitary *GH1* and *GHRHR* mRNAs were the lowest on Day 7, increased during adolescence to reach a peak on Day 40, and finally declined to an intermediate level from Day 90 to Day 250 (Figure 1c,d). Pituitary *SSTR2* mRNA expression consistently increased with age after birth (Figure 1e).

### 2.2. Dynamic Expression of miRNAs in the Pituitary

**Figure 2:**
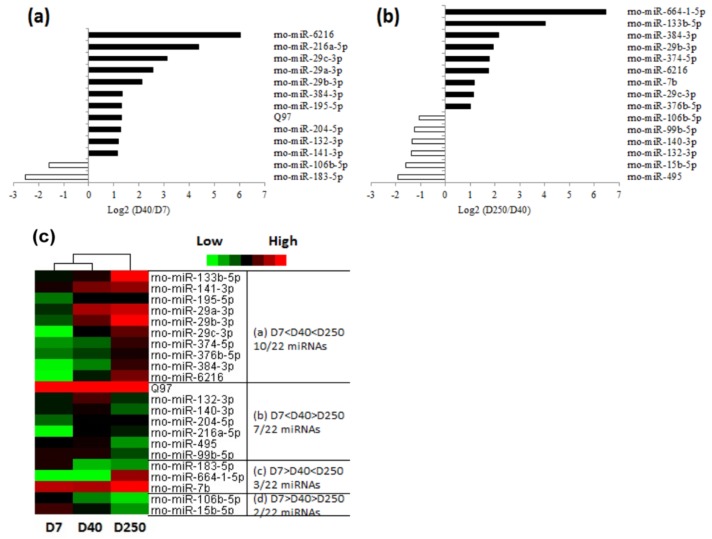
The comparisons and heat map of the differentially expressed microRNAs (miRNAs) among the different ages. (**a**) Differentially expressed miRNAs between Day 7 and Day 40. (**b**) Differentially expressed miRNA between Day 250 and Day 40. Results were reported as fold change in specific miRNA expression. Each bar corresponds to the expression fold difference, calculated as log _2_ [(D40 signal)/(D7 signal)] or log _2_ [(D250 signal)/(D40 signal)], of miRNA listed in the figure. The fold change is over 2. (**c**) Heat map of the differentially expressed miRNAs. Each row represents the relative level of expression of a single miRNA, and each column indicates the expression levels for a single sample. The red or green color indicates relatively high or low expression, respectively.

From the microarray assay, we detected 411 unique miRNAs in the pituitary, including 388 previously known rat miRNAs and 23 novel candidates (Table S1). After performing normalization and a quality assessment, the miRNA expression on Day 40 versus Day 7 in the pituitaries and Day 250 versus Day 40 were compared, respectively. It was found that the expression level of 13 miRNAs (11 miRNAs were upregulated, while 2 were downregulated) changed greater than 2-fold on Day 40 compared to that on Day 7 in the pituitaries (Figure 2a); 15 miRNAs (nine miRNAs were upregulated, while six were downregulated) changed on Day 250 compared to that on Day 40 (Figure 2b). It is notable that there were six overlapping miRNAs (miR-29b-3p, miR-29c-3p, miR-384-3p, miR-132-3p, miR-106b-5p, and miR-6216) among the total 28 differentially expressed miRNAs. Taken together, these findings identified 22 pituitary miRNAs differentially expressed with advancing age. As shown in Figure 2c, the expression patterns of the pituitary miRNA with age shared four typical categories: (1) the expression level increased from Day 7 to Day 40 and Day 250; (2) the expression level increased from Day 7 to Day 40, but decreased from Day 40 to Day 250; (3) the expression level decreased from Day 7 to Day 40, but increased from Day 40 to Day 250; and (4) the expression level decreased from Day 7 to Day 40 and Day 250.

### 2.3. Target and Pathway Analysis of Differentially Expressed miRNAs

Each miRNA can regulate numerous target genes and, therefore, has the potential to modulate multiple pathways. To explore which targets and pathways may be regulated by these differentially expressed miRNAs, we used IPA to predict the targets co-expressed with them in the pituitary and revealed the molecule type, gene location, and canonical pathways associated with each miRNA (Table S2). The targets of the differentially expressed miRNAs were most prominently predicted to function as kinases, enzymes, and transcription regulators. The pathway results revealed that as many as 511 pathways might be affected by the various miRNAs with different expression in aging pituitaries (Table S3). The data presented in Table S3 demonstrate various pathways that are regulated by several of the age-related miRNAs. The value of each row represents the number of miRNAs involved in each of the pathways. The results of the analysis revealed that 15 of the differentially expressed miRNAs related to age may participate in the signaling pathways closely associated with a pituitary function (Table 1).

**Figure 3:**
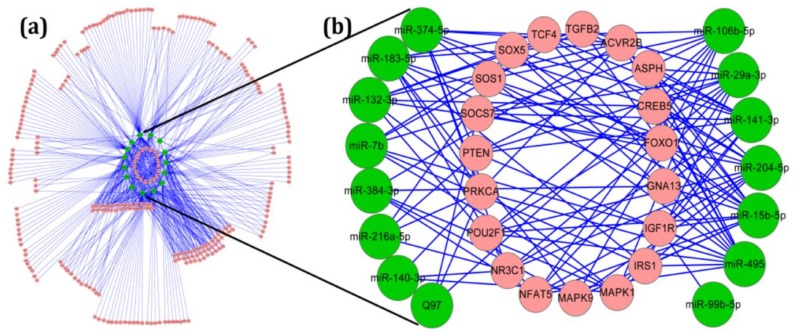
A network of miRNAs and their predicted targets participate in the pathways involved in pituitary function. (**a**) All of the miRNA–gene interaction bipartite networks participate in the pathways involved in pituitary function. (**b**) miRNA–gene bipartite network interactions that represent potential target genes with five or more focus miRNAs. The green ellipses indicate miRNAs, and the red ellipse indicates the predicted targets.

The bioinformatics targets of these miRNAs were then graphed using Cytoscape to demonstrate the interactions between pituitary-function-associated miRNAs and their potential mRNA targets (Figure 3). According to the miRNA–gene interaction bipartite network, some single transcripts may be targeted by single or multiple miRNAs (Figure 3a), and there were 19 transcripts predicted to be targeted by 5 or more focus miRNAs (Figure 3b). However, it is notable that there were no differentially expressed miRNAs directly targeting GH1 in the bioinformatics analysis.

### 2.4. Validation of the Pituitary miRNAs via qRT-PCR

**Figure 4:**
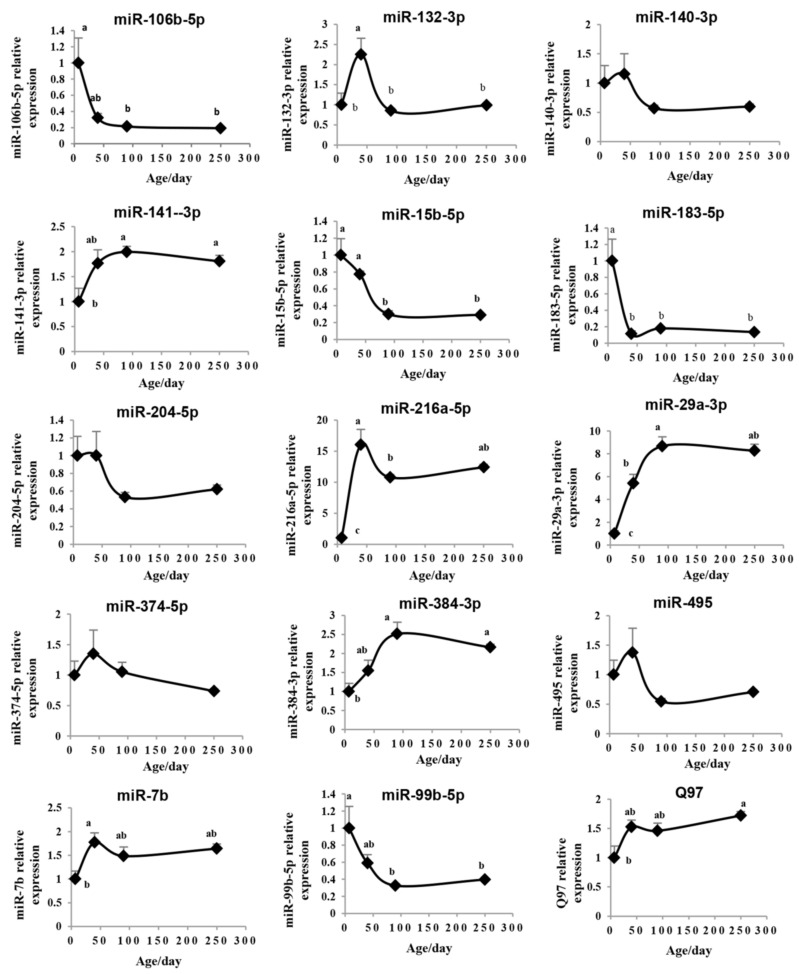
QRT-PCR detection of the temporal expression patterns of the candidate miRNAs. The expressions of 15 miRNAs on rat pituitaries of different ages (Day 7, Day 40, Day 90, and Day 250) were detected by qRT-PCR. The values are expressed as the means ± SEM. Means with different letters were significantly different (*n* = 8; *p* < 0.05; ANOVA; Tukey-HSD).

**Figure 5:**
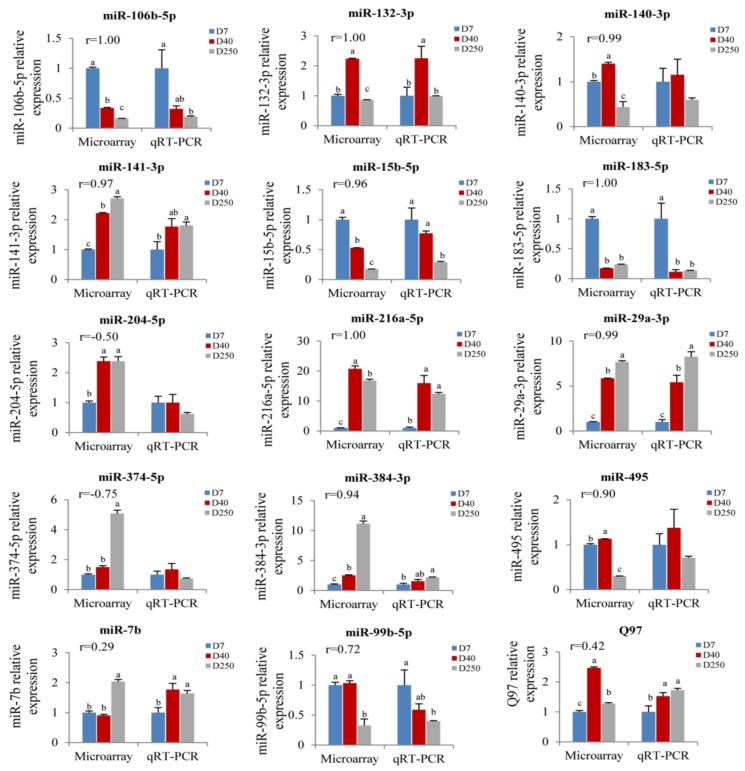
Validation of the microarray results by real-time PCR. Expression level of 15 miRNAs was detected by microarray (left) and real-time PCR (right). All data are represented as the means ± SEM. A Pearson correlation coefficient (*r*) was performed to determine the consistency between the real-time PCR and microarray data. The expression abundance on Day 7 was normalized to 1. Statistical significance was determined by a one-way ANOVA followed by a Tukey-HSD test. *p* < 0.05 was considered as statistically significant, and the column of each group with different letters and same letters, respectively, represent statistically significant and insignificant. The column with double letters means that this set of data is not significant with other two sets of data.

To verify and evaluate the reliability of the results obtained from the microarray analysis, we selected 15 miRNAs to be tested using a real time quantitative polymerase chain reaction (qRT-PCR) assay (Figure 4). These miRNAs were selected from the differentially expressed miRNAs in the IPA analysis (Figure 3b). Then, a Pearson correlation analysis was used to analyze the correlation between the results of a chip and quantitative PCR detection of 15 candidate miRNAs on Days 7, 40, and 250, respectively (Figure 5). The qRT-PCR results of 5 miRNAs (miR-204-5p, miR-374-5p, miR-7b, miR-99b-5p, and Q97) were inconsistent with the microarray, while the other 10 out of 15 miRNAs exhibited a good correlation (*r* > 0.90) with the results of the microarray. Although the expression of miR-384-3p seemed to be inconsistent on Day 250, it had the same uptrend with the microarray.

### 2.5. Determination of the Functional miRNA Target Pairs

**Figure 6:**
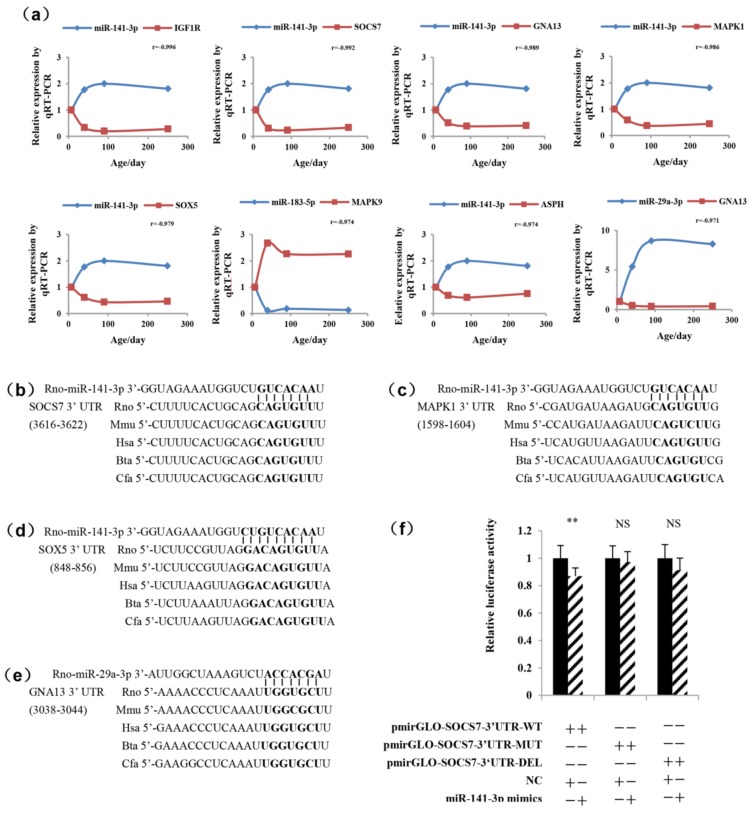
Temporal expression patterns and conservation analysis of negatively correlated miRNA/mRNA gene pairs. (**a**) The expression levels of the eight miRNAs/mRNA gene pairs were detected by real-time PCR. The blue curves and red curves, respectively, represent miRNAs and their predicted genes expressions. All data are represented as the mean ± SEM. A Pearson correlation coefficient (*r*) was performed to determine the correlation between the miRNAs and mRNAs. The seed regions of miR-141-3p and the seed-recognizing sites in the *SOCS7* 3′UTR (**b**), *MAPK1* 3′UTR (**c**), and *SOX5* 3′UTR (**d**) and the seed regions of miR-29a-3p and the seed-recognizing sites in the *GNA13* 3′UTR (**e**) are indicated in boldface type. All nucleotides in the seed-recognizing sites were completely conserved in several species. *Rno*, *Rattus norvegicus*; *Mmu*, *Mus musculus*; *Hsa*, *Homo sapiens*; *Bta*, *Bos taurus*; *Cfa*, *Canis familiaris*. (**f**) Relative luciferase activities were detected after the Hela cells were co-transfected with pmirGLO-*SOCS7*-3′UTR-(WT/MUT/ DEL) plasmid and negative control (NC) or miR-141-3p mimics. Results are presented as means ± SEM (*n* = 8). **, *p* < 0.01; NS, not significant (*p* > 0.05).

The information gathered from IPA allowed us to identify 19 candidate genes (Figure 3b) which could serve as possible miRNA targets via qRT-PCR (Figure S1). Furthermore, we analyzed the expression pattern of the 19 genes and their predicted potential miRNAs and calculated the correlation coefficient (Table S4). In general, miRNAs were found to inhibit mRNA expression [29]. In the present study, we selected the top eight miRNA/mRNA pairs showing negative correlations in Table S6. The eight negative miRNA/predicted gene pairs (Figure 6a) were miR-141-3p/*IGF1R*, miR-141-3p/suppressor of cytokine signaling 7(*SOCS7*), miR-141-3p/ G protein subunit alpha 13 (*GNA13*), miR-141-3p/*MAPK1*, miR-141-3p/sex determining region Y (*SPY*)-box 5 (*SOX5*), miR-141-3p/ aspartate-beta-hydroxylase (*ASPH*), miR-183-5p/*MAPK9*, and miR-29a-3p/*GNA13*. Then, TargetScan was used to analyze the conservation of the target relationship among species (Figure 6b–e), and the miR-141-3p/*SOCS7*, miR-141-3p/*MAPK1*, miR-141-3p/*SOX5*, and miR-29a-3p/*GNA13* miRNA/predicted gene pairs were conserved. To confirm the result predicted by the bioinformatics method, miR-141-3p and its potential target *SOCS7* were chosen for further validation by a luciferase reporter assay (Figure 6f). The results showed that the luciferase activity of the transfected cells with miR-141-3p mimics and pmirGLO-*SOCS7*-3′UTR-WT was significantly decreased compared with the cells co-transfected with negative control (NC) and pmirGLO-*SOCS7*-3′UTR-WT. The luciferase activity in the construct of mutations and the deletion in the 3′UTR of *SOCS7* did not show any change.

### 2.6. miR-141-3p Negatively Regulates Rat Pituitary GH Expression

**Figure 7:**
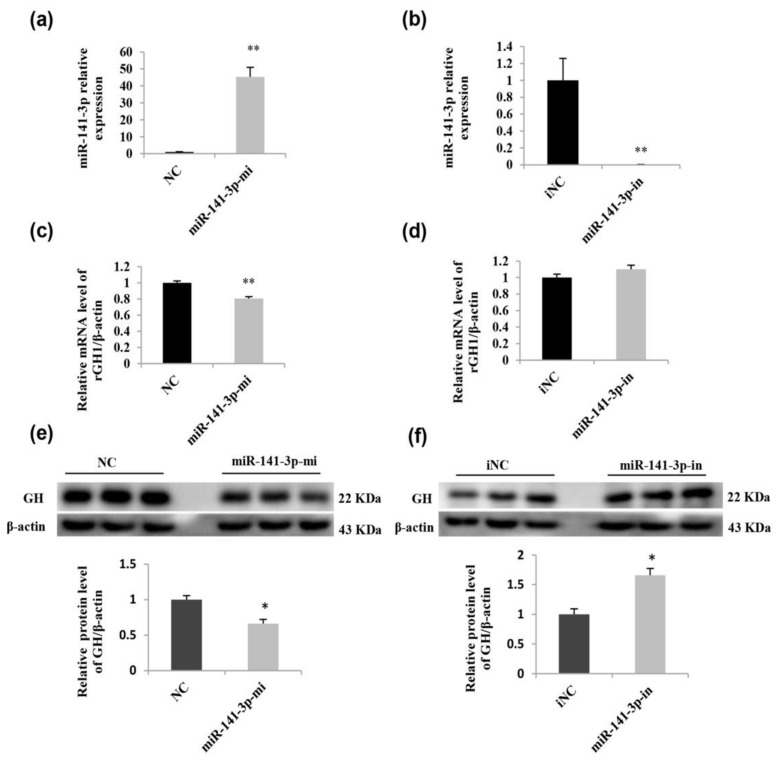
miR-141-3p negatively regulates growth hormone (GH) mRNA and protein expression. GH3 cells were separately transfected with 100 pM miR-141-3p mimic and miR-141-3p inhibitor. Forty-eight hours later, GH3 cells were collected. (**a**,**b**) Analysis of miR-141-3p levels by qRT-PCR. (**c**,**d**) Analysis of *GH1* mRNA levels by qRT-PCR. (**e**,**f**) Analysis of GH protein levels by Western blot and quantitative analysis of the Western blot results. Results are presented as the means ± SEM (*n* = 3). *, *p* < 0.05, **, *p* < 0.01, (*t* test). iNC, inhibitor negative control.

To verify whether miR-141-3p or miR-29a-3p regulates GH expression in GH3 cells, the mimics or inhibitors were transfected into GH3 cells. Then, we found that only miR-141-3p had an effect on GH expression. Transfecting the mimic effectively increased the cellular level of miR-141-3p (Figure 7a), while the inhibitor reduced the level of the corresponding miRNA (Figure 7b). The qRT-PCR results revealed that miR-141-3p overexpression in GH3 cells resulted in a reduction of *GH1* mRNA (Figure 7c). A Western blot analysis of GH indicated that the miR-141-3p mimic significantly inhibited GH at the protein level (Figure 7e), whereas the miR-141-3p inhibitor significantly increased the GH protein level (Figure 7f). These results suggest that miR-141-3p is involved in GH regulation.

## 3. Discussion

In the present study, we conducted an analysis of rat growth from birth through adolescence to adulthood by measuring the body weight and serum IGF-1 levels as well as detecting the expression of genes related to growth, including *GH1*, *GHRHR*, and *SSTR2*. All of the test indexes were markedly age-dependent. The miRNAs have been reported to participate in pituitary and GH regulation; however, information regarding miRNA alteration with age remains limited. Thus, we profiled the miRNA expression in the rat pituitary at three distinct ages (Day 7, Day 40, and Day 250) simultaneously via a miRNA microarray. In summary, we validated and confirmed 22 differentially expressed miRNAs in the rat pituitary. In addition, most of the altered miRNAs were identified to participate in signaling pathways associated with pituitary functionality. There were 15 miRNAs that were predicted to target 19 candidate genes by IPA. To determine the actual positive and functional miRNA/target pairs, the mRNA expression of the miRNAs and genes was detected by qRT-PCR and the correlation coefficient was calculated. The miR-141-3p/*SOCS7*, miR-141-3p/*MAPK*1, miR-141-3p/*SOX5*, and miR-29a-3p/*GNA13* pairs were all negatively correlated. Finally, we chose miR-141-3p for further study and preliminarily found that miR-141-3p inhibits pituitary GH expression. Although the miRNA profile had been analyzed in the pituitary of several species [30,31,32], no studies to date have analyzed miRNA expression in the pituitaries of rats from birth throughout development. Our study presented a microarray analysis of the pituitary miRNAs of rats from three distinct ages and explored the potential role of pituitary miRNAs in regulating GH.

The growth curve of the rats in this study reflected a general growth rate that parallels the results of the *Sprague-Dawley* (SD) male rat growth chart on the Taconic Biosciences website, excepting that the plateau that occurs by Day 90 is a little early. The other three ages reflected different typical stages of rat growth. Day 7 is the neonatal period, Day 40 is the start time of a rapid growth period, and Day 250 is in a platform of growth associated with a stable weight. Therefore, investigations of miRNA expression patterns in the three time points may provide useful information about the miRNA temporal expressions in the pituitary. It has been reported that GH levels are extremely elevated in immature mammals and decline with age [33,34]. In our study, the alteration of *GH1* mRNA levels with age was primarily consistent with previous reports except the data on Day 90. Iruthayanathan et al. [35] found that female rat pituitary GH mRNA levels decrease progressively with age from 3–4 months to an ~50% decrease by 9 months, while our study showed a sharp decrease by Day 90 (~3 months) in male rats. It is deduced that the discrepancy in pituitary GH mRNA is possibly due to sex [36,37,38]. GH secretion and synthesis are regulated by growth hormone-releasing hormone (GHRH) and somatostatin, which play roles through their related receptors GHRHR and SSTR. Reed et al. [39] analyzed pituitary somatostatin receptor 1–5 expression during rat development and found that pituitary *SSTR2* mRNA increased markedly and progressively with advancing age after birth. The study also detected a similar changing profile related to the mRNA of *SSTR2*. Korytko et al. [40] determined the ontogeny of rat *GHRHR* gene expression and found that the *GHRHR* mRNA levels increased during the first 30 days of life and then declined with age. A consistent result that the expression of *GHRHR* mRNA increased from Day 7 to Day 40 was revealed. However, again, the level of *GHRHR* mRNA on Day 90 was low too early [35,40]. Due to several discrepancies on Day 90 compared with previous reports, this time point was not selected for further microarray analysis.

Recent reports have suggested that miRNAs might play a role in the aging process. For example, the expression of the miRNA lin-4 decreased with advancing age, whereas the overexpression of lin-4 extended the lifespan of Caenorhabditis elegans [41]. Maes et al. [42] used murine livers from different time points as a model to screen miRNA expression and found that miR-669c and miR-709 were gradually increased from middle-aged animals at 18–33 months, while miR-93 and miR-214 were significantly upregulated only in extremely old livers. As the age-related miRNAs in the pituitary have not been widely studied, in this study, we analyzed the age-related miRNAs expression in the male rat pituitary and also studied the role of miR-141-3p on GH regulation. Except for miR-141-3p, we also detected that mR-29, miR-183, miR-374, and miR-204 were differentially expressed with advancing age, which was also observed in other study [32]. It is notable that miR-141-3p and the miR-29 family had also been reported to be age-related in other tissues or species [43,44,45,46]. Thus, it can be deduced that the miR-29 family and miR-141 may not only be regulated by age but may also play an important role in the aging process. Additionally, other new age-related miRNAs (e.g., miR-384-3p, miR-132-3p, miR-106b-5p, and miR-6216) in the rat pituitary were identified in our study, indicating that they may have special functions in the pituitary tissues. In the research, we have studied the relationship between miR-141-3p and GH, but the roles of the new identified age-related miRNAs still require further studies.

Identifying the specific target genes of miRNAs is a critical step to investigate miRNA functionality [47,48]. IPA not only predicts the potential target genes but also generates the relevant tissue, which may help locate tissue-specific miRNA targets [49]. In this study, IPA was employed to predict the potential targets of differentially expressed miRNAs in the pituitary with age. These results provide useful information for the further analysis of miRNA function in the pituitary. From the IPA and TargetScan analysis, *SOCS7*, *MAPK1*, and *SOX5* were predicted targets of miR-141-3p. We found miR-141-3p negatively regulates GH. Since GH is not the direct target of miR-141-3p, it is most likely that miR-141-3p regulates *GH1* expression via other targets. The relations between miR-141-3p and its target genes as well as their roles in GH regulation need to be verified in subsequent experiments. Furthermore, we identified no differentially expressed miRNAs directly targeting *GH1*. At present, there still no validation for miRNAs targeting *GH1*. Actually, we also used the whole set of miRNA expression profile data to predict miRNAs targeting *GH1* by a miRanda analysis, and miR-543-5p, miR-199a-5p, and a novel miRNA Q62 were predicted to target *GH1*.

In conclusion, this study surveyed the miRNA expression in the pituitaries of rats throughout the entire lifespan using a microarray and provided novel insight into the alteration of miRNA expression during both development and aging. We found that 22 miRNAs were differentially expressed in the pituitary with age, some of which were predicted to be involved in the signaling pathways related to pituitary function. Moreover, we analyzed pairs of miRNAs/target genes. Finally, in vitro tests revealed that miR-141-3p inhibited GH expression in the pituitary. Taken together, our results shed new light on miRNA expression and GH regulation in the pituitary and indicate that altered miRNAs are potential meaningful biomarkers for studying the pituitary during postnatal development.

## 4. Materials and Methods

### 4.1. Animals and Sample Preparation

Specific pathogen-free male *Sprague-Dawley* (SD) rats were purchased from the Guangdong Medical Laboratory Animal Center (Foshan, China). The experimental animals were categorized into four different groups according their age: 7-day-old newborn rats (group D7; *n* = 8), 40-day-old adolescent rats (group D40; *n* = 8), 90-day-old young adult rats (group D90; *n* = 8), and 250-day-old adult rats (group D250; *n* = 8). All animal procedures were conducted under the protocol (SCAU-AEC-2013-0416, 16 April 2013) approved by Institutional Animal Care and Use Committee (IACUC) of South China Agricultural University. All rats were weighed and then were anesthetized with diethyl ether and killed via a rapid decapitation. The blood was collected from the jugular vein in a 2 mL polyethylene tube and centrifuged at 3000 rpm for 20 min at 4 °C. After centrifugation, the serum was stored at −80 °C for preservation. The pituitaries were removed and stored at −80 °C until processing. The growth curve of the rats was determined by measuring the body weight.

### 4.2. Serum IGF-1 Concentration Assay

IGF-1 was extracted from serum to eliminate IGF-1 binding proteins (IGFBPs) following the procedure reported by Niu et al. [50]. Briefly, 20 μL of the serum samples was added to 180 μL of the extraction solution (875 mL/L absolute ethanol in 2 mol/L HCl) in a 0.5 mL polyethylene microfuge tube, incubated at room temperature for 30 min, and subsequently centrifuged at 4 °C (10,000 rpm for 2 min). A portion of the clear supernatant (140 μL) was neutralized with 70 μL of 0.855 mol/L Tris base (pH 11.0), mixed, and incubated at room temperature for 30 min. After centrifugation at 4 °C (10,000 rpm for 2 min), the supernatant was decanted and allowed to reach room temperature. The final dilution factor was 1:30. The serum IGF-1 concentration was measured by Radio Immunoassay (RIA) using commercial kits from Nine Tripods Medical & Bioengineering Co. Ltd. (Tianjin, China) as previously described [51]. All measurements were made within 3 days after collecting the samples.

### 4.3. RNA Extraction and Quantitative Real-Time PCR Analysis

The total RNA from the pituitary was extracted using TRIzol reagent (Invitrogen, Carlsbad, CA, USA) following the manufacturer’s instructions. The quality of the RNA was monitored by spectrophotometry (ND-2000, NanoDrop Inc., Wilmington, DE, USA), and the 28 S and 18 S were examined by electrophoresis. DNase I (Promega, Madison, WI, USA) was used to remove DNA contamination. Reverse transcription of mRNAs and miRNAs was performed with poly-T primers using the M-MLV reverse transcriptase system (Promega, Madison, WI, USA) and One Step PrimeScript^®^ miRNA cDNA Synthesis Kit (TaKaRa, Tokyo, Japan) following the manufacturer’s protocol, respectively. The cDNA was diluted 5-fold with ddH_2_O. Quantitative real-time PCR was performed using STRATAGENE Mx3005P sequence detection system (Aglient Technologies, Santa Clara, CA, USA) as follows: denaturation (95 °C/10 min), PCR amplification and quantification (95 °C/15 s, 56–60 °C/15 s, and 72 °C/40 s) with a single fluorescence measurement at the end of the elongation step, repeated for 40 cycles. Each reaction contained 2 μL of cDNA, 0.3 μmol/L of each primer, 10 µL of 2× FastStart Universal SYBR Green Master (Rox) (Roche, Basel, Switzerland), and water to make up the final volume of 20 μL. The primers used for the miRNAs, genes, U6, and β-actin are listed in Table S5. The data were analyzed by comparing the miRNAs and genes at the mRNA level with β-actin and U6 expression, respectively, for each rat, and then subjecting the values to statistical analysis. All qRT-PCR reactions were performed twice.

### 4.4. MicroRNA Microarray Assay

According to the results of the growth curve, serum IGF-1 level, and pituitary mRNAs expression, three (D7, D40, and D250) of the four groups were selected for the chip test. A total of 827 miRNAs, including 727 rat miRNAs from miRBase 20.0 and 100 randomly designed novel candidates, which refer to sequences homologous to human or mouse miRNAs, were used (Table S6). For each group, the total RNA from eight pituitaries was pooled together as a sample for the miRNA expression profile analysis, which was conducted using custom microarrays at LC Sciences (Houston, TX, USA). Four replicate microarrays were carried out. The microarray assay was performed as described by Marsh et al. [52] and Li et al. [53]. The data were analyzed by first subtracting the background and normalizing the signals using a Lowess filter (Locally-weighted Regression) [54]. The replicated miRNAs were averaged, and miRNAs with intensities >2000 in all samples were chosen for calculating the normalization factor. The expression data were normalized using Median normalization. After normalization, differentially expressed miRNAs were identified via the Fold Change of two-fold filtering. To create a hierarchical clustering map, we used Cluster 3.0 with Java TreeView (available online: http://rana.lbl.gov/EisenSoftware.htm) [55].

### 4.5. Tissue-Specific miRNA Target Prediction and Exploration of the Involved Pathways

Identification of the putative target genes for all of the differentially expressed miRNAs was performed using the miRNA Target Filter in IPA (available online: http://www.ingenuity.com/). The IPA tool has a miRNA target filter function which relies on experimentally validated interactions from the TarBase database [56], miRecords (available online http://mirecords.biolead.org/), TargetScan (available online http://www.targetscan.org/), and manually curated miRNA-related findings within the IPA knowledgebase. IPA also generates the relevant diseases, location, molecule type, pathway, species, and tissue/cell line where the predicted miRNA targets are found. As mRNAs cannot be affected by miRNAs that are not co-expressed with them [49], transcripts not expressed in the pituitary were filtered out from the list of potential targets. In addition, the predicted targets were subjected to pathway exploration using IPA.

### 4.6. Cell Lines and Cell Transfection

The rat pituitary cell line GH3, obtained from the American Type Culture Collection (ATCC), was maintained in F-12K Medium containing 15% horse serum and 2.5% fetal bovine serum (FBS) at 37 °C in a humidified atmosphere of 5% CO_2_. Rattus norvegicus miR-141-3p mimic (forward: 5′-UAACACUGUCUGGUAAAGAUGG-3′ and reverse: 5′-AUCUUUACCAGACAGUGUUAUU-3′) and Rattus norvegicus miR-141-3p inhibitor (5′-CCAUCUUUACCAGACAGUGUUA-3′) were commercially synthesized from Shanghai GenePharma (Shanghai, China). The stable negative control (NC) (forward: 5′-UUCUCCGAACGUGUCACGUTT-3′ and reverse, 5′-ACGUGACACGUUCGGAGAATT-3′) and inhibitor NC (iNC) (5′-CAGUACUUUUGUGUAGUACAA-3′) were used as negative controls. One day before transfection, the cells were seeded onto Poly-l-Lysine (PLL)-coated six-well plates at 2 × 106 cells/well in the absence of antibiotics. For transfection, the cells were grown to 50–80% confluence in the culture plates. The cells were then transfected with 100 pmol/well oligonucleotides by Lipofectamine 2000 (Invitrogen, Carlsbad, CA, USA) according to the manufacturer’s protocol. At 6 h post-transfection, the cells were washed once with 1× PBS, cultured in complete culture medium, and grown for 48 h before collection. The miR-141-3p mimic, inhibitor, and their respective negative controls, NC and iNC, were obtained from Shanghai GenePharma (Shanghai, China).

### 4.7. Western Blot Analysis

GH3 cells were lysed in RIPA buffer (BioTeke, Beijing, China) containing 1 mmol/L protease inhibitor PMSF (Sigma, St. Louis, MO, USA) followed by 12,000× *g* centrifugation at 4 °C for 15 min. Supernatant protein concentrations were determined using a BCA Protein Assay Reagent Kit (Thermo Fisher Scientific, Waltham, MA, USA). After boiling for 5 min, the samples (30 μg each) were subjected to 5–10% SDS-polyacrylamide gel electrophoresis (PAGE) at 80 V for 20 min and 100 V for 70 min using Tris-glycine running buffer. The separated proteins were transferred onto PVDF membranes (Millipore, Billerica, MA, USA) by electroblotting in a transfer buffer (25 mmol/L Tris base, 192 mmol/L glycine, and 10% methanol, pH 8.3). The membranes were blocked in 5% BSA in TBST buffer (20 mmol/L Tris, 500 mmol/L NaCl, 0.05% Tween-20, pH 7.6) for 2 h at room temperature and then incubated overnight at 4 °C with primary antibodies. The next day, the membranes were washed six times for 5 min each with TBST buffer followed by incubation with horseradish-peroxidase-labeled secondary antibodies for 1 h at room temperature. Anti-GH (MAB1566) were purchased from R&D Systems (Minneapolis, MN, USA). Anti-β-actin(bsm-33139M), anti-rabbit IgG(bs-0295G), and anti-mouse IgG(bs-0296G) were obtained from Bioss (Beijing, China). Immunoreactivity was detected using the ECL Plus chemiluminescence detection kit (Beyotime, Shanghai, China) in a FluorChem M system (Cell Biosciences, San Leandro, CA, USA). The density of the bands was analyzed using the Image JSoftware (Tanon, Shanghai, China).

### 4.8. Luciferase Reporter Assay

The Dual-Luciferase reporter genes were constructed using the pmirGLO Vector (Promega, Madison, WI, USA) and the 3′UTR sequence of rat *SOCS7*. The 3′UTR of *SOCS7* contains the highly conserved binding sites of miR-141-3p, and the sequence containing the binding sites (113 bp) is as follows, the bold and underline sequence represents the binding sites.

5′-TCGAGCATCAACACAGCTGTCCTTACACTAAATCTGTGCTATTGCATACA

TGTAGCCATCTTTCTTTTCACTGCAG**CAGTGTT**TATCAGTAGTTCAAAATGATTTATTTGCTT-3′.

Furthermore, the 3′UTR sequence was inserted into the pmirGLO Vector with XhoI and XbaI double digestion to construct the recombinant dual-luciferase reporter vector pmirGLO*-SOCS7*-3′UTR-WT. Meanwhile, a plasmid containing the mutant *SOCS7* 3′UTR, pmirGLO-*SOCS7*-3′UTR-MUT, was generated by mutating the core sequence of the miR-141-3p binding sites through DNA synthesis (Sangon Biotech Co. Ltd., Shanghai, China), and the sequence is as follows, the bold and underline sequence represents the mutational sites.

5′-TCGAGCATCAACACAGCTGTCCTTACACTAAATCTGTGCTATTGCATACA

TGTAGCCATCTTTCTTTTCACTGCAG**ACAGAGG**TATCAGTAGTTCAAAATGATTTATTTGCTT-3′.

Similarly, a plasmid containing the deleted *SOCS7* 3′UTR, pmirGLO-*SOCS7*-3′UTR-DEL, was generated by deleting the core sequence of miR-141-3p binding sites through DNA synthesis, and the sequence is as follows:

5′-TCGAGCATCAACACAGCTGTCCTTACACTAAATCTGTGCTATTGCATACA

TGTAGCCATCTTTCTTTTCACTGCAGTATCAGTAGTTCAAAATGATTTATTTGCTT-3′.

Hela cells (4 × 10^4^ per well) were plated in a 96-well plate. At 60–70% confluency, cells were transfected with 3 pmol miR-141-3p mimics/NC and 100 ng pmirGLO-*SOCS7*-3′UTR-WT/pmirGLO-*SOCS7*-3′UTR-MUT/pmirGLO-*SOCS7*-3′UTR-DEL. Cells were collected 48 h after transfection, and luciferase activity was measured with the Dual-GLO luciferase reporter assay system (Promega, Madison, WI, USA) according to the manufacturer’s guideline.

### 4.9. Statistical Analysis

The statistical analysis was performed using SPSS Statistics Software. A Student’s *t*-test was used to compare the differences between the two groups, and a one-way ANOVA followed by a Tukey’s HSD test was used to compare the differences among multiple groups. The differences were considered significant at *p* < 0.05. Correlation is a technique for investigating the relationship between two quantitative, continuous variables [57]. A Pearson’s correlation coefficient (*r*), known as the product-moment coefficient of correlation, was determined for each pair of miRNAs and the corresponding predicted target gene using Microsoft Excel [58,59]. The data pertaining to the miRNA expression obtained by microarray and qRT-PCR were also analyzed with a Pearson’s correlation coefficient.

## Figures and Tables

**Table 1:** Pituitary-function-related pathways regulated by aging-associated miRNAs.

Pathway	Count of miRNAs
Calcium Signaling	15
Cyclic adenosine monophosphate (cAMP)-mediated Signaling	15
Externally regulated kinases/ mitogen-activated protein kinase (ERK/MAPK) Signaling	15
Glucocorticoid Receptor Signaling	15
Gonadotropin-releasing hormone (GNRH) Signaling	15
Growth Hormone Signaling	15
Insulin-like growth factor 1 (IGF-1) Signaling	15
Insulin Receptor Signaling	15
Protein Kinase A Signaling	15
Retinoic acid receptor (RAR) Activation	15
Wnt/β-catenin Signaling	15
Corticotropin Releasing Hormone Signaling	14
cAMP-response element binding protein (CREB) Signaling in Neurons	14
G-Protein Coupled Receptor Signaling	14

## Supplementary Materials

Supplementary materials can be found at http://www.mdpi.com/1422-0067/19/7/2058/s1.

## Abbreviations

GH	Growth hormone
miRNA	MicroRNA
mRNA	Messenger RNA
RT-PCR	Reverse transcriptase quantitative polymerase chain reaction
3′UTR	3′ Untranslated region
GHRHR	Growth hormone-releasing hormone receptor
IPA	Ingenuity pathway analysis
SSTR2	Somatostatin receptor 2
