# Supplementary material for: Age-Related Changes in MicroRNA in the Rat Pituitary and Potential Role in GH Regulation
Source: Int J Mol Sci. 2018 Jul 15;19(7):2058. doi: 10.3390/ijms19072058 (PMC6073141; doi:10.3390/ijms19072058)
Supplement: Supplementary file 1 [file ijms-19-02058-s001.zip › supplementary files/Figure S1.pdf]

Figure S1

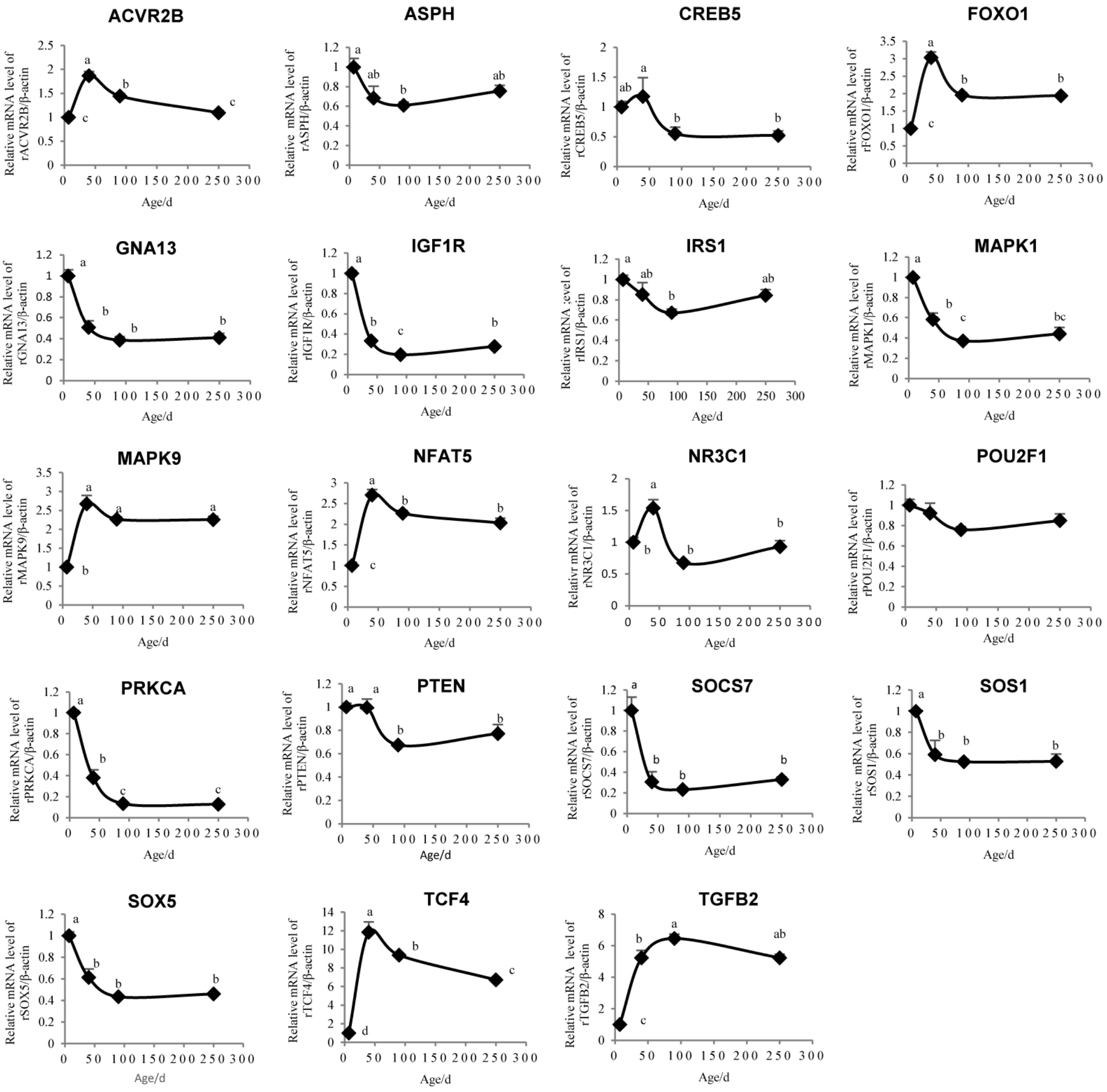

Figure S1. Real time quantitative polymerase chain reaction (qRT-PCR) detection of the temporal expression patterns of the candidate mRNAs. The expressions of 19 candidate mRNAs were detected by qRT-PCR. The values are expressed as the means ± SEM. Means with different letters were significantly different (n = 8; $p< 0.05$; ANOVA; Tukey-HSD).
